# Bilateral idiopathic retinal vasculitis following coxsackievirus A4 infection: a case report

**DOI:** 10.1186/s12886-017-0523-2

**Published:** 2017-07-19

**Authors:** Izumi Mine, Manzo Taguchi, Yutaka Sakurai, Masaru Takeuchi

**Affiliations:** Department of Ophthalmology, National Defense Medical College, 3-2 Namiki, Tokorozawa City, Saitama, 359-8513 Japan

**Keywords:** Coxsackievirus, Idiopathic retinal vasculitis, Multifocal obstructive retinal vasculitis, Virus infection

## Abstract

**Background:**

Coxsackieviruses are members of a group of viruses called the enteroviruses, which may cause respiratory and gastrointestinal symptoms, erythema, meningoencephalitis, myocarditis, pericarditis, and myositis. Unilateral acute idiopathic maculopathy caused by coxsackievirus A16 has been associated with hand, foot, and mouth disease, but only a few reports describe retinitis associated with coxsackievirus serotype B3 or B4. We report a case of bilateral multifocal obstructive retinal vasculitis that developed after coxsackievirus A4 infection.

**Case presentation:**

A 60-year-old woman was referred to our department with bilateral visual disturbance that developed following flu-like symptoms. At the initial examination, best corrected visual acuity was 20/200 in the right eye and 20/50 in the left eye. The critical flicker frequency (CFF) was 23 Hz in the right eye and 27 Hz in the left eye. Fine white keratic precipitates with infiltrating cells were presented in the anterior chamber of both eyes, and multifocal retinal ischemic lesions were observed in the macula and posterior pole of both eyes. The retinal lesions corresponded with scotomas observed in Goldmann visual field test. On spectral domain-optical coherence tomography (SD-OCT), retinal lesions were depicted as hyper-reflective regions in the inner retina layers in both eyes, and disruption of ellipsoid line in the left eye., Fluorescein angiography exhibited findings indicative of multifocal obstructive retinal vasculitis. The patient had a history of current hypertension treated with oral therapy and glaucoma treated with latanoprost eye drops. Blood test for coxsackievirus antibody titers revealed that A4, A6, A9, B1, B2, B3, and B5 were positive (titers: 8–32). Abdominal skin biopsy of necrotic tissue suggested vascular damage caused by coxsackievirus. The general symptoms improved after 6 weeks, and the multifocal retinal ischemic lesions were partially resolved with residual slightly hard exudates. Only coxsackievirus A4 antibody titer increased from 4 to 32-fold after 14 months. However, hyper-reflective regions and disruption of the inner retinal layers on SD-OCT persisted especially in the right eye, and residual paracentral scotoma was observed in the right eye.

**Conclusion:**

The present case suggests that coxsackievirus A4 causes bilateral multifocal obstructive retinal vasculitis with irreversible inner retinal damage.

## Background

Coxsackieviruses belong to the enterovirus genus of the family *Picornaviridae*, and are classified into two groups: A and B. Group A coxsackieviruses cause flaccid paralysis, whereas group B coxsackieviruses cause spastic paralysis. Regarding concurrent coxsackievirus infection in the posterior segment of the eye, many reports have described an association of hand, foot, and mouth disease caused by coxsackievirus A16 with unilateral acute idiopathic maculopathy (UAIM) [1–8]. However, there are few reports of retinitis associated with coxsackievirus, and only serotype B3 or B4 was reported [9–12]. Herein, we report a patient with reduced visual acuity caused by coxsackievirus A4-induced bilateral multifocal obstructive retinal vasculitis, which was observed by multimodal imaging.

## Case presentation

**Fig. 1:**
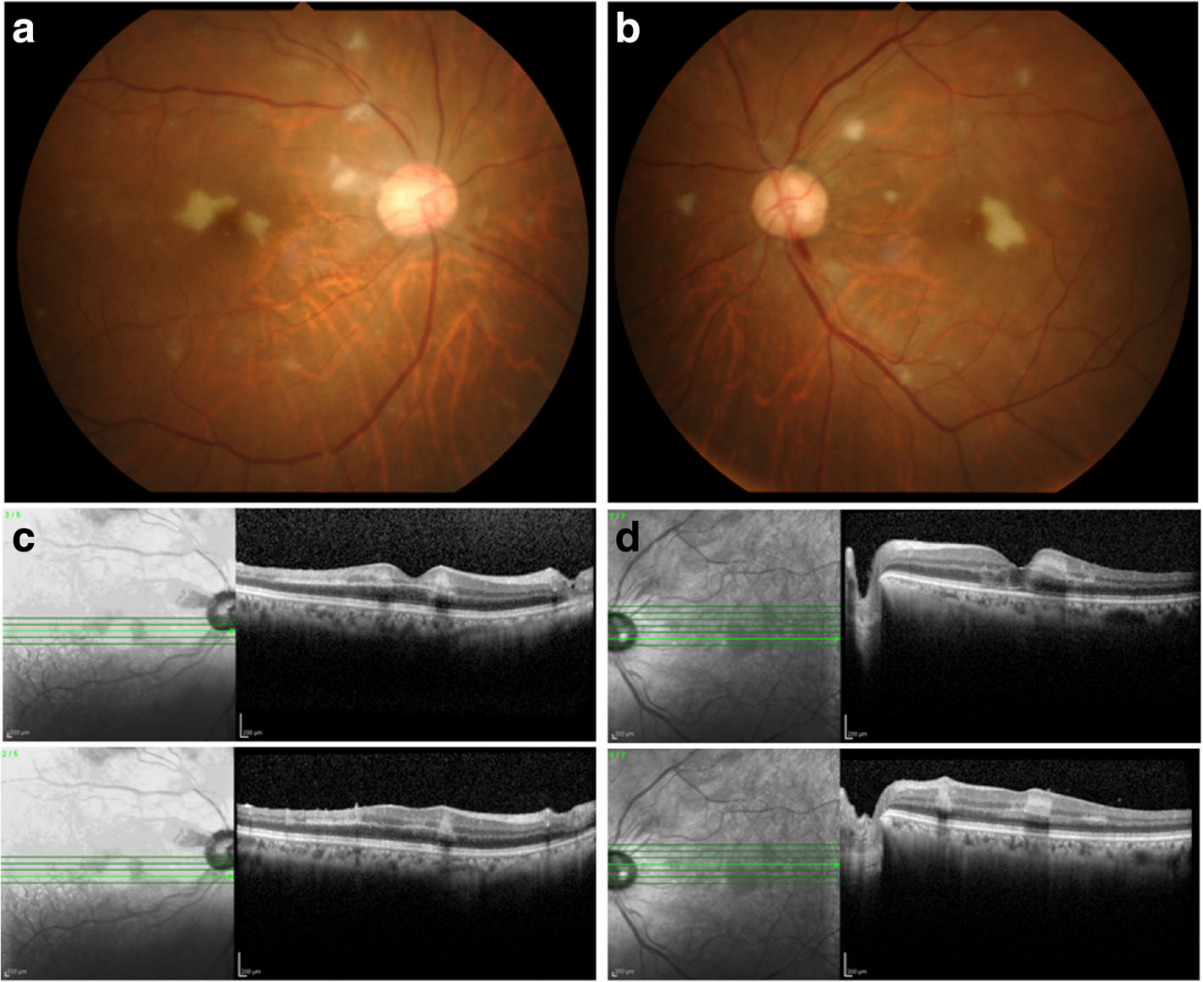
Fundus photographs and SD-OCT images at the initial examination**. a** and **b** Fundus photographs show multifocal ischemic lesions around the macula and posterior pole in both *right* (**a**) and *left* (**b**) eyes. **c** and **d** SD-OCT reveals hyper-reflective regions in the inner retina layers in both right (**c**) and left (**d**) eyes, and disruption of ellipsoid line in the left eye

**Fig. 2:**
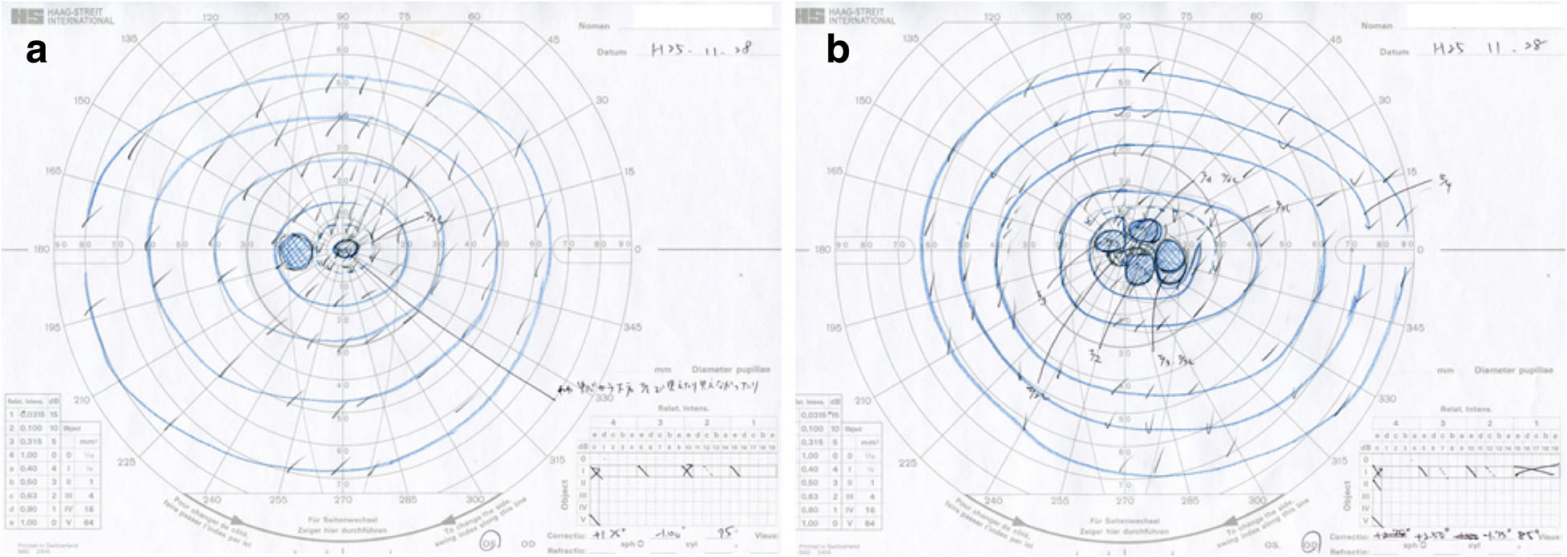
Goldmann visual field test after 2 weeks. Goldmann visual field test conducted after 2 weeks in the *left* (**a**) and *right* (**b**) eyes shows central and paracentral scotomas

**Fig. 3:**
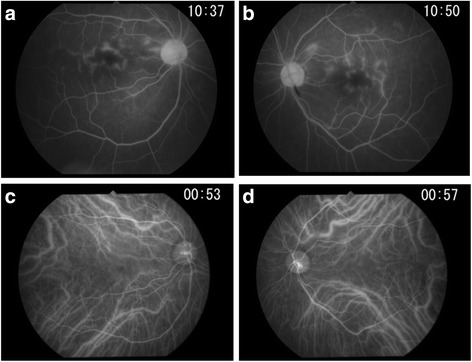
Fluorescein and indocyanine green angiography**. a** and **b** Fluorescein angiography reveals filling defect in the affected part of the retina with leakage of fluorescence dye from the surrounding retinal blood vessels in both *right* (**a**) and *left* (**b**) eyes. **c** and **d** Indocyanine green angiography reveals no abnormalities in both right (**c**) and left (**d**) eyes

A 60-year-old female had flu-like symptoms from November 1, 2013, and subsequently developed fever, articular pain, retroauricular lymph node swelling, erythema, and dizziness with gait disturbance 4 days later. The patient was admitted to the Department of Internal Medicine at National Defense Medical College Hospital for detailed examination. Nine days after onset, the patient complained of bilateral visual disturbance and was referred to our department. She has a history of current hypertension treated with oral therapy and glaucoma treated with latanoprost eye drops. At the initial ophthalmological examination, best corrected visual acuity was 20/200 in the right eye and 20/50 in the left eye, and intraocular pressure was normal in both eyes. The critical flicker frequency (CFF) was 23 and 27 Hz for the right and left eye, respectively, and a relative afferent pupil defect was noted in the right eye. Slit lamp examination showed fine white keratic precipitates with infiltrating cells in the anterior chamber in both eyes. Funduscopy revealed multifocal retinal ischemic lesions around the macula and posterior pole in both eyes. (Fig. 1a and b). Scotoma areas corresponding with the retina lesions were observed by Goldmann visual field test (Fig. 2). On SD-OCT, multifocal white retinal lesions were depicted as hyper-reflective regions in the inner retina layers of both eyes, and disruption of ellipsoid line was observed in the left eye (Fig. 1c and d). Fluorescein angiography (FA) showed bilateral filling defects corresponding to the retinal lesions, which were surrounded by dye leakage from retinal capillaries (Fig. 3a and b). Indocyanine green angiography depicted no abnormalities in both eyes (Fig. 3c and d). Brain and orbital MRI were also performed, but the result was not particular.

On day 12 after onset of ocular symptoms, aqueous humor sample was collected and tested using multiplex polymerase chain reaction (PCR) for human herpes virus (HHV) 1–8, toxoplasma, toxascaris, bacterial 16srDNA, and fungal 28srDNA, all of which were negative. On the same day, treatment with betamethasone and phenylephrine tropicamide eye drops was initiated. Blood test for coxsackievirus antibody titers revealed that A4, A6, A9, B1, B2, B3, and B5 were positive (titers: 8–32; Table 1). An abdominal skin biopsy of necrotic tissue suggested vascular damage caused by coxsackievirus. On the other hand, since the patient fulfilled the diagnostic criteria for polymyalgia rheumatic (PMR), oral corticosteroid (15 mg/day prednisolone) was initiated on November 25, 2013.

**Table 1:** Changes in serum coxsackievirus antibody titers determined by neutralization test (NT)

Coxsackievirus	2013/11/12	2013/11/25	2014/5/1	2015/1/13
A4 (NT)	8	16	16	32
A6 (NT)	8	8	<4	<4
A9 (NT)	32	16	16	4
A16 (NT)	<4	<4	<4	<4
B1 (NT)	–	4	<4	<4
B2 (NT)	–	8	4	4
B3 (NT)	–	16	8	4
B4 (NT)	–	<4	<4	<4
B5 (NT)	–	8	8	8
B6 (NT)	–	<4	<4	<4

**Fig. 4:**
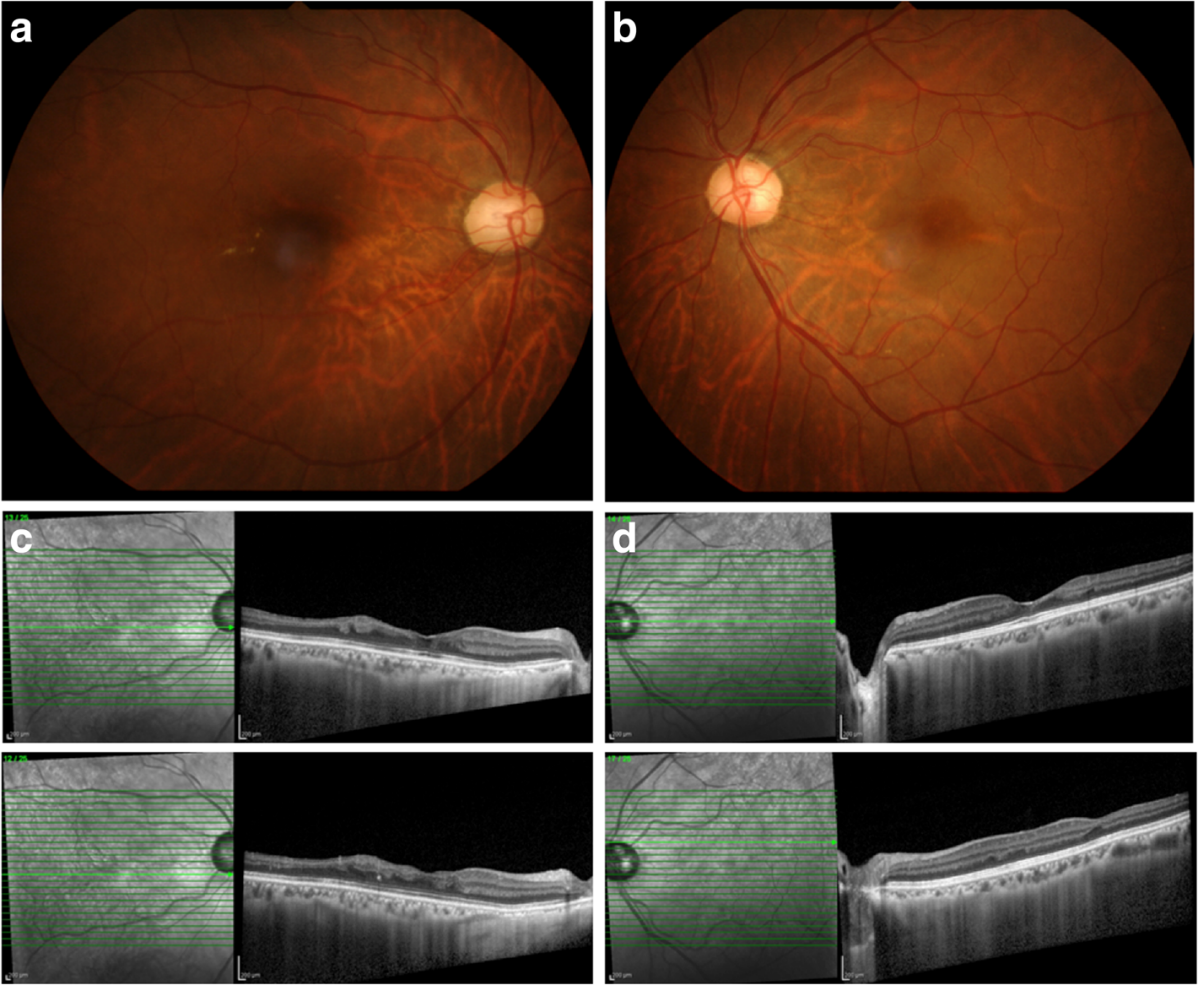
Fundus photographs and SD-OCT image after 14 months**.** (**a** and **b**) The fundus photographs show slightly hard exudates in both right (**a**) and *left* (**b**) eyes. (**c** and **d**) Hyper-reflective regions and disruption of the inner retinal layers persist in both right (**c**) and left (**d**) eyes, especially in the *right* eye

**Fig. 5:**
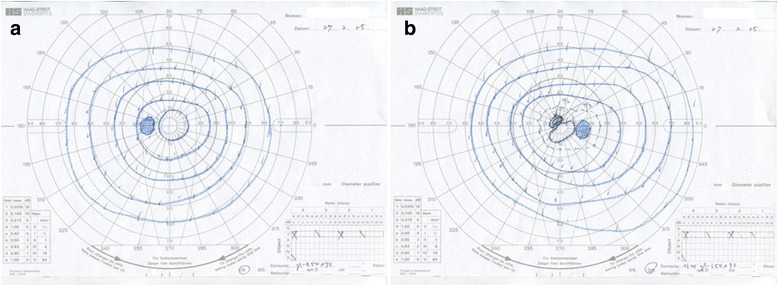
Goldmann visual field test after 14 months**.** Goldmann visual field test conducted after 14 months in the left (**a**) and right (**b**) eyes shows disappearance of central scotoma in the left eye, but persistence of paracentral scotoma in the right eye

The general symptoms improved after 6 weeks, and the multifocal retinal ischemic lesions were partially resolved and residual exudates were slightly hard. (Fig. 4a and b). However, hyper-reflective regions and disruption of the inner retinal layers on SD-OCT persisted, especially in the right eye (Fig. 4c and d). The steroid dose was tapered, and from March 2015, the patient was put under follow-up observation with prednisolone 1 mg/day. After 14 months, the coxsackievirus A4 antibody titer increased by 32-fold (Table 1). Corrected visual acuity was 20/25 in the right eye and 20/15 in the left eye, and residual paracentral scotoma was observed in the right eye (Fig. 5).

## Conclusions

Although coxsackievirus is apparently a rare cause of multifocal obstructive retinal vasculitis, it should be considered in the appropriate clinical setting. Since it has become more convenient to detect several HHV by multiplex PCR using ocular fluid, reports of HHV-related ocular diseases are increasing [13]. However, such convenient tests are not available for the detection of coxsackieviruses, and currently diagnosis still depends on clinical signs such as flu-like symptoms and changing antibody titers after resolution of the infection. When molecular biological techniques become a standard method for the detection of coxsackieviruses, more cases of coxsackievirus-associated ocular disease may be diagnosis with a heightened level of suspicion.

In the present case, obstructive retinal vasculitis appeared to be induced via immune response to virus infection, similar to the clinical conditions of acute retinal necrosis caused by HHV1, HHV2 or HHV3, and the skin necrosis probably resulted from impaired blood flow because of the same mechanism. Several cases of retinitis associated with coxsackievirus have been reported [8–11]. Takeuchi et al. [9] reported a case of coxsackievirus B4 infection causing iridocyclitis and obstructive retinal vasculitis. Other case reports described coxsackievirus-associated macular lesion with posterior segment inflammation, similar to UAIM [8, 10, 11]. However, the serotype of coxsackievirus in all the above cases was “B”, and most of the lesions were reversible. Coxsackievirus A4-associated retinitis leads to bilateral irreversible retinal damage has not been reported previously.

In summary, we have described the clinical features of coxsackievirus A4-associated retinitis and bilateral obstructive vasculitis causing irreversible retinal damage. Abnormalities on FA and SD-OCT suggest the critical role of inflammation at the level of inner retinal layers. SD-OCT was especially valuable for monitoring partial changes of the entire retinal layers.

## Abbreviations

CFF: Critical flicker frequency
HHV: Human herpes virus
PCR: Polymerase chain reaction
PMR: Polymyalgia rheumatic
SD-OCT: Spectral domain-optical coherence tomography
UAIM: Unilateral acute idiopathic maculopathy

## References

[1] Beck AP, Jampol LM, Glaser DA, Pollack JS. Is coxsackievirus the cause of unilateral acute idiopathic maculopathy? Arch Ophthalmol 2004;122(1):121-123. Epub 2004/01/14. doi: 10.1001/archopht.122.1.121. PubMed PMID: 14718310. 10.1001/archopht.122.1.121 14718310

[2] Meyerle CB, Yannuzzi LA. Acute positive titers of antibody to coxsackievirus in acute idiopathic maculopathy. Retin Cases Brief Rep. 2008;2(1):34-35. Epub 2008/01/01. doi: 10.1097/01.iae.0000243065.91330.e0. PubMed PMID: 25389612. 10.1097/01.iae.0000243065.91330.e0 25389612

[3] Hughes EH, Hunyor AP, Gorbatov M, Ho IV. Acute idiopathic maculopathy with coxsackievirus infection. Retin Cases Brief Rep. 2012;6(1):19-21. Epub 2012/01/01. doi: 10.1097/ICB.0b013e3181f7f7ee. PubMed PMID: 25390701. 10.1097/ICB.0b013e3181f7f7ee 25390701

[4] Jung CS, Payne JF, Bergstrom CS, Cribbs BE, Yan J, Hubbard GB, 3rd, et al. Multimodality diagnostic imaging in unilateral acute idiopathic maculopathy. Arch Ophthalmol. 2012;130(1):50–56. doi: 10.1001/archophthalmol.2011.359. PubMed PMID: 22232475; PubMed Central PMCID: PMCPMC4116109. 10.1001/archophthalmol.2011.359 PMC4116109 22232475

[5] Demirel S, Batioglu F, Ozmert E, Batioglu F. Unilateral acute maculopathy related to hand, foot, and mouth disease: OCT and fluorescein angiography findings of a very rare disease. Eur J Ophthalmol 2014;24(1):131-133. Epub 2013/05/11. doi: 10.5301/ejo.5000288. PubMed PMID: 23661540. 10.5301/ejo.5000288 23661540

[6] Vaz-Pereira S, Macedo M, De Salvo G, Pal B. Multimodal imaging of exudative maculopathy associated with hand-foot-mouth disease. Ophthalmic Surg Lasers Imaging Retina. 2014;45 Online:e14–e17. doi: 10.3928/23258160-20140331-01. PubMed PMID: 24695047. 10.3928/23258160-20140331-01 24695047

[7] Tandon M, Gupta A, Singh P, Subathra G (2016). Unilateral hemorrhagic maculopathy: An uncommon manifestation of hand, foot, and mouth disease. Indian J Ophthalmol.

[8] Haamann P, Kessel L, Larsen M. Monofocal outer retinitis associated with hand, foot, and mouth disease caused by coxsackievirus. Am J Ophthalmol. 2000;129(4):552–553. doi: http://dx.doi.org/10.1016/S0002-9394(99)00440-7. 10.1016/s0002-9394(99)00440-7 10764878

[9] Takeuchi M, Sakai J, Usui M (2003). Coxsackievirus B4 associated uveoretinitis in an adult. Br J Ophthalmol.

[10] Kadrmas E, Buzney S. Coxsackievirus B4 as a cause of adult chorioretinitis. Am J Ophthalmol. 1999;127(3):347–349. doi: http://dx.doi.org/10.1016/S0002-9394(98)00322-5. 10.1016/s0002-9394(98)00322-5 10088751

[11] Hirakata K, Oshima T, Azuma N. Chorioretinitis induced by coxsackievirus B4 infection. Am J Ophthalmol 1990;109(2):225-227. Epub 1990/02/15. PubMed PMID: 2154107. 10.1016/s0002-9394(14)75993-8 2154107

[12] Hollsten JE, McClintock M, Samy H, Peden M, Kay CN. Fulminant chorioretinitis and papillitis secondary to coxsackievirus B presenting as acute posterior multifocal placoid pigment epitheliopathy with positive response to intravenous immunoglobulin. Retin Cases Brief Rep 2013;7(3):225-231. Epub 2013/07/01. doi: 10.1097/ICB.0b013e31828993c6. PubMed PMID: 25391111. 10.1097/ICB.0b013e31828993c6 25391111

[13] Sugita S, Ogawa M, Shimizu N, Morio T, Ohguro N, Nakai K, et al. Use of a comprehensive polymerase chain reaction system for diagnosis of ocular infectious diseases. Ophthalmology 2013;120(9):1761-1768. doi: 10.1016/j.ophtha.2013.02.020. PubMed PMID: 23664179. 10.1016/j.ophtha.2013.02.020 23664179

